# TRAIL promotes hepatocellular carcinoma apoptosis and inhibits proliferation and migration via interacting with IER3

**DOI:** 10.1186/s12935-020-01724-8

**Published:** 2021-01-20

**Authors:** Shihai Liu, Jing Qiu, Guifang He, Weitai He, Changchang Liu, Duo Cai, Huazheng Pan

**Affiliations:** 1 Medical Animal Lab, The Affiliated Hospital of Qingdao University, Qingdao, 266000 China; 2 Department of Stomatology, Qingdao Municipal Hospital, Qingdao, 266071 China; 3 Department of Clinical Laboratory, The Affiliated Hospital of Qingdao University, Qingdao, 266000 China

**Keywords:** Tumor necrosis factor-related apoptosis-inducing ligand, Wnt pathway, Hepatocellular carcinoma

## Abstract

Tumor necrosis factor-related apoptosis-inducing ligand (TRAIL) can induce substantial cytotoxicity in tumor cells but rarely exert cytotoxic activity on non-transformed cells. In the present study, we therefore evaluated interactions between TRAIL and IER3 via co-immunoprecipitation and immunofluorescence analyses, leading us to determine that these two proteins were able to drive the apoptotic death of hepatocellular carcinoma (HCC) cells and to disrupt their proliferative and migratory abilities both in vitro and in vivo. From a mechanistic perspective, we determined that TRAIL and IER3 were capable of inhibiting Wnt/β-catenin signaling. Together, these results indicate that TRAIL can control the pathogenesis of HCC at least in part via interacting with IER3 to inhibit Wnt/β-catenin signaling, thus indicating that this TRAIL/IER3/β-catenin axis may be a viable therapeutic target in HCC patients.

## Introduction

Hepatocellular carcinoma (HCC) and chronic liver disease (CLD) are increasingly prevalent throughout the world, accounting for 800,000 and 2 million deaths worldwide each year, respectively [1–3]. CLD can additionally drive the development of hepatic scarring, fibrosis, and HCC. Non-alcoholic fatty liver disease (NAFLD) can also promote HCC development over the course of many years even in the absence of cirrhosis [4]. HCC prevalence in China is particularly high, accounting for ~ 55% of global HCC cases [5]. While several diagnostic and therapeutic approaches to detecting and treating HCC have been developed, at present the 5-year survival rate for those with this disease remains poor and has not improved substantially [6]. It is therefore vital that novel diagnostic biomarkers and therapeutic targets be identified in order to improve the targeted treatment of HCC patients.

TNF-related apoptosis-inducing ligand (TRAIL) is a protein that can induce the apoptotic death of many types of tumor cells, leading many researchers to attempt to develop TRAIL-receptor agonists as a form of anti-cancer therapy [7, 8]. Following its secretion from natural killer (NK) cells, TRAIL is capable of binding to cell surface death receptors (DR4 and DR5) [9], after which the pro-form of caspase-8 is recruited to the Fas-associated death domain (FADD) adaptor protein and is activated, thereby initiating apoptotic signaling cascades that culminate in caspase-3 activation and cell death [10]. To date, however, DR4/5 agonists and agonistic antibodies specific for these TRAIL receptors have failed to achieve clinical anti-cancer efficacy in phase II clinical trials [11, 12]. This lack of efficacy is generally associated with factors including off-target systemic toxicity, poor agonist bioavailability in tumor tissues, the fact that recombinant TRAIL has a short plasma half-life, and the fact that tumor cells often downregulate DR4/5 or upregulate anti-apoptotic proteins to survive [11]. Future research is thus essential in order to overcome these limitations so as to better apply TRAIL-based therapeutics to the treatment of different forms of cancer.

Given that attempts to administer recombinant proteins have failed in this therapeutic context, systemic or intratumoral TRAIL gene transfer therapy has been identified as a potentially viable alternative approach to cancer treatment [13–15]. Several different vectors have been designed and used alone or together with combination chemotherapy to treat cancer by facilitating TRAIL gene transfer with high efficiency, minimal immunogenicity, and little off-target toxicity [16–18]. Gene transfer approaches such as polyethyleneimine (PEI) and other cationic polymers, dendrimers, and liposomes have all been identified as potent approaches to efficient TRAIL gene transfer capable of driving tumor cell apoptosis [16, 19].

Immediate early response gene (IER3), also called IEX-1, is expressed in a wide range of human tissues, including liver tissues [20]. Previous studies have shown that IER3 expression in cancer is significantly reduced [21], and overexpression of IER3 could promote the apoptosis of cancer cells and enhance its sensitivity to chemotherapeutic drugs [22]. In the field of radiation sensitivity of cells, recent studies have found that IER3 gene regulation sequences have radiation induced characteristics [23].

Herein, we employed co-IP approaches to demonstrate an interaction between TRAIL and IER3. We then employed MTT, wound healing, Transwell, and flow cytometry assays to explore the impact of TRAIL and IER3 on tumor cell apoptosis and migratory activity. Furthermore, we utilized an HCC xenograft model system and additional Western blotting to explore the mechanisms whereby these two proteins slow HCC growth in vivo. Overall, our results reveal that TRAIL and IER3 can induce the apoptotic death of HCC cells via influencing the Wnt signaling pathway.

## Materials and methods

### Cell culture, transfection, and adenoviral preparation

HEK293, HepG2 and Hep3B cells were obtained from the American Type Culture Collection (ATCC), while BEL7402 and SMMC7721 cells were obtained from the Type Culture Collection of the Chinese Academy of Sciences (Shanghai, China). Huh7 cells were obtained from Procell Life Science & Technology Co., Ltd. (Wuhan, China). All cells were cultured in RPMI-1640 containing 10% FBS (Gibco, life technologies, California, USA) and penicillin/streptomycin (Solarbio, beijing China) at 37 ℃ in a 5% CO_2_ incubator.

In the indicated experiments, cells were transfected with control, TRAIL, or IER3 overexpression vectors using plasmids prepared by GeneChem (Shanghai, China). Briefly, cells were plated in 6-well plates and grown overnight until 70–80% confluent, at which time Lipofectamine 3000 (Thermo Fisher Scientific, Waltham, MA, USA) was used to transfect cells. At 8 h post-transfection, media was replaced and cells were cultured for an appropriate time prior to downstream experimentation.

The pAdEasy-1 vector in *E. coli* BJ5183 was used to prepare recombinant adenoviruses, as in prior studies [24] An appropriate multiplicity of infection was determined by comparing total numbers of infected cells to total plaque-forming units, with duplicate samples being used to titrate prepared adenoviruses to titers of 1 × 10^11^ pfu/ml.

### MTT assay

Cells were plated in 96-well plates (10,000/well) for an experimentally-determined period of time, after which 10 μL of MTT (5 mg/mL) was added per well followed by an additional 2 h incubation at 37 °C. Media was then removed, and 100 μL of DMSO was added to each well. Absorbance at 490 nm was then assessed with a microplate reader (model 680, Bio-Rad Laboratories, Hercules, CA). All sample and control samples were analyzed in sextuplicate.

### Co-immunoprecipitation (co-IP) and western blotting

Interactions between IER3 and TRAIL were assessed via co-IP assays. Cells were first lysed using RIPA buffer after being washed with PBS. Lysates were then mixed for 12 h with appropriate primary antibodies linked to protein A/G sepharose beads (Roche, shanghai, China) at 4 °C following protein G sepharose bead pre-clearing. SDS-PAGE was then used to separate precipitated immunocomplexes, and separated proteins were transferred to PVDF membranes (Millipore, Bedford, MA, USA). Blots were then blocked for 2 h using 5% non-fat milk in TBST, followed by an overnight incubation with antibodies specific for TRAIL (1:1,000; Santa-Cruz Biotechnology, Santa Cruz, CA, USA), IER3 (1:1,000; Sigma, St. Louis, MO), Myc tag (1:1,000; Sigma, St. Louis, MO), and Flag tag (1:1,000; Cell Signaling, Danvers, MA) at 4 °C. Blots were next washed thrice, followed by incubation for 2 h with appropriate HRP-linked secondary anti-rabbit and anti-mouse antibodies (1:1,000; Pierce, USA). A Bio-Rad ChemiDoc Imaging System (Bio-Rad, California, USA) was then employed for protein band detection.

### GST pulldown

*E. coli* BL21(DE3) were initially cultured overnight, after which they were diluted 100-fold in fresh Lysogeny broth (LB) containing 100 mg/L ampicillin, and were cultured at 37 °C until racing an OD600 of 0.4–0.6 Recombinant GST-fusion protein expression was induced by adding 0.1 mM isopropyl-β-d-thiogalactoside for 12 h at 24 °C. Protein was then purified, immobilized on glutathione-agarose (Sigma-Aldrich, St. Louis, MO), and separated via SDS-PAGE to gauge protein amounts. In addition, we expressed Flag-IER3 in E. coli BL21(DE3) and employed a Flag-specific mAb to purify this protein with an agarose gel (Sino biological, China). GST-fusion proteins were then incubated with a Flag-Select affinity gel precoated with Flag-IER3 for 12 h at 4 °C with constant agitation. Beads were then washed extensively with RIPA buffer, after which protein elution and Western blotting were performed.

### Immunofluorescent staining

Huh7 and SMMC7721 HCC cells were cultured in individual wells of 8-well chamber slides (Nunc). Following cell attachment, cells were transfected using pcDNA3-TRAIL and/or pcDNA3-IER3, followed by an additional 48 h incubation. Cells were then washed thrice using PBS, fixed for 15 min with 4% paraformaldehyde (PFA), permeabilized in 0.1% Triton X-100 in PBS/3% BSA and blocked for 1 h using 1% BSA in PBST. Cells were then incubated overnight with an appropriate primary antibody, after which they were washed thrice using PBS and incubated for 30 min with AF488 goat anti-rabbit IgG (Cell Signaling, Danvers, MA, 1:1,000) or AF555 goat anti-mouse IgG (Cell Signaling, Danvers, MA, 1:1,000) at 37 °C. After PBS washing, cells were then stained using DAPI prior to imaging with a Leica TCS SP5 confocal microscope (Leica Microsystems).

### Apoptosis and colony formation assays

A FACSCalibur™ flow cytometer (BD) was used to evaluate cellular apoptosis after HCC cells had been stained with an Annexin V-PE and 7-AAD Apoptosis Detection kit (Beyotime, Jiangsu, China) based on provided directions. Briefly, cells expressing TRAIL and/or IER3 were harvested during the logarithmic growth phase, stained, and analyzed, with FlowJo being used for data analysis.

For colony formation assays, roughly 1,000 cells/group were resuspended in 10 mL of RPMI-1640 and were cultured for 10 days until visible colonies were evident. Cells were then fixed for 15 min with 4% PFA, and were stained for 20 min using 0.5% crystal violet. All colonies containing > 50 cells were then counted under 100 × magnification to quantify the colony-forming abilities of different experimental groups.

### Wound healing assay

Cellular migration was assessed with a wound healing assay. Briefly, cells were cultured in 6-well plates until 90–95% confluent. Cells were then infected for 24 h with adenoviruses encoding TRAIL and/or IER3, after which a wound was generated in the cell monolayer using a sterile micropipette tip. Free cells were washed away, and the baseline wound was imaged. Cells were incubated for 24 h in RPMI-1640 containing 10% FBS, after which the wound was again imaged at appropriate time points, with cellular migration into the wound site over time thereby being quantified.

### In vitro migration assay

A Transwell 24-well filter insert (Corning, 8.0 μm pore size) system was used to evaluate HCC cell migration. Briefly, inserts were treated for 30 min with 200 μL of serum-free RPMI-1640 at 37 °C, after which this media was removed and 600 μL of fresh media supplemented with 20% FBS was added into the lower chamber. Into the upper chamber, cells that had been infected 24 h previously with Ad5-TRAIL and/or Ad5-IER3 were added (1 × 10^5^/well) in 100 μL of serum-free media. Plates were then incubated for 24 h, after which methanol was used to fix the upper chamber for 30 min, followed by crystal violet staining for an additional hour. Non-migrated cells were then removed from the upper chamber with a cotton swab, and the remaining cells were imaged and counted using an inverted ECLIPSE Ts2 microscope (Nikon, Japan).

### In vivo xenograft model

A murine xenograft model system was used to evaluate the impact of TRAIL/IER3 synergistic effect on HCC tumor growth. All animal experiments were consistent with the National Guidelines for Housing and Care of Laboratory Animals, and were approved by the Institutional Animal Care and Use Committee of the Institute of Medicine, The Affiliated Hospital of Qingdao University. Briefly, female athymic mice (BALB/c nude, 6–8 weeks old, 18–20 g) from Vital River Laboratories (Beijing, China) were subcutaneously implanted with Huh7 or SMMC7721 cells (2 × 10^6^ cells in 100 μL PBS). Tumor growth was monitored every 2 days, with tumor volume (V) being determined as follows: V (mm^3^) = 0.5 × a × b × b, where a and b correspond to the longest diameter of the tumor and the diameter perpendicular to a, respectively.

When tumors had grown to 50 mm^3^ in size, animals were randomly assigned to Ad5-EGFP, Ad5-TRAIL, Ad5-IER3, and Ad5-TRAIL/Ad5-IER3 groups. Animals in these groups received intratumoral injections of 5 × 10^8^ PFU of the indicated adenovirus in 50 μl PBS once every two days over an 8-day period. After 24 days, mice were then sacrificed via CO_2_ asphyxiation, and tumors were collected, weighed, imaged, and subjected to pathological analyses [25].

### TOP/FOP-Flash luciferase reporter analysis

Wnt/β-catenin signaling pathway activity was quantified via a TOP/FOP-Flash luciferase reporter assay, with the pRL-SV40 vector being used as an internal control. Control and NC cells were co-transfected with pRL-SV40 and TOP/FOP flash (Promega, WI, USA). Cells in all groups were co-transfected with pRL-SV40 and TOP/FOP flash, and then treated them for 24 h with LiCl (20 mM), with those in the TRAIL + IER3 group additionally being infected with the TRAIL and IER3-encoding adenoviruses. Luciferase activity was then quantified using a Dual-Luciferase Reporter Assay System (Promega, WI, USA), with the TOP/FOP ratio being used to quantify Wnt/β-catenin signaling pathway activity following normalization of firefly luciferase activity to Renilla luciferase activity.

we transfected Huh7 cells with the appropriate reporters, and then treated them for 24 h with LiCl (20 mM), after which cells were infected with Ad5-TRAIL and Ad5-IER3 or Ad5-EGFP. LiCl treatment was confirmed to increase the TOP/FOP ratio of these HCC cells (P < 0.01), consistent with Wnt/β-catenin signaling pathway activation.

### Immunohistochemical staining

The Medical Ethics Committee of Shanghai, China approved the present study. Tissue microarrays that contained 90 HCC tumor tissue samples and 90 paracancerous normal tissue samples (HLivH180Su14) were obtained from Outdo Biotech Co. Ltd. (Shanghai, China), and clinicopathological data pertaining to the patients incorporated into this microarray (including age, sex, tumor stage, tumor grade, tumor size, HBsAg, HBcAb, HCV status, and follow-up data) were assessed (Table 1). In addition, diagnosis and staging of the HCC tumor samples within these microarrays were validated internally based upon clinical and pathological evaluation in accordance with the 8th edition of the American Joint Committee on Cancer (AJCC) staging system. No patients included in this study had undergone adjuvant chemotherapy treatment prior to tumor resection.

**Table 1:** Correlation between IER3 expression and clinicopathologic characteristics of HCC patients

Characteristics IER3	No. of cases (%)	IER3 expreesion			
		Low or none, no. cases	High, no. cases	Statistic (c^2^)	p value
Age					
< 50	37 (41.1)	14	23	1.509	0.219
≥ 50	53 (58.9)	27	26		
Gender					
Male	80 (88.9)	37	43	0.14	0.708
Female	10 (11.1)	4	6		
HBsAg					
Positive	70 (77.8)	29	41	2.84	0.092
Negative	19 (21.1)	12	7		
HBcAb					
Positive	80 (88.9)	35	45	1.985	0.159
Negative	7 (7.8)	5	2		
HCV					
Positive	1 (1.1)	0	1	0.861	0.353
Negative	86 (95.6)	40	46		
Liver cirrhosis					
Yes	80 (88.9)	38	42	0.09	0.419
No	9 (10.0)	3	6		
Total bilirubin (mmol/L)					
< 21	75 (83.3)	36	39	0.717	0.397
≥ 21	14 (15.6)	5	9		
ALT (U/L)					
< 60	70 (77.8)	29	41	2.84	0.092
≥ 60	19 (21.1)	12	7		
GGT (U/L)					
< 40	30 (33.3)	9	21	4.702	*0.030*
≥ 40	59 (65.6)	32	27		
AFP (ng/L)					
< 20	36 (40.0)	17	19	0.067	0.795
≥ 20	54 (60.0)	24	30		
Cirrhotic nodules					
Single	10 (11.1)	5	5	0.653	0.765
Multiple	80 (88.9)	36	44		
Vascular invasion					
Yes	21 (23.3)	12	9	1.545	0.214
No	58 (64.4)	24	34		
Tumor number					
Single	79 (87.8)	36	43	0	0.994
Multiple	11 (12.2)	5	6		
Tumor size					
< 5 cm	55 (61.1)	16	39	15.458	* < 0.001*
≥ 5 cm	35 (38.9)	25	10		
Recrudescence					
Yes	49 (54.4)	27	22	3.952	*0.047*
No	41 (45.6)	14	27		
AJCC TNM stage					
I stage	58 (64.4)	24	34	1.392	0.499
II stage	29 (32.2)	15	14		
III stage	3 (33.3)	2	1		

P values were calculated using chi-square test. Italics numbers indicate significant differences (P < 0.05)

*HCC* hepatocellular carcinoma, *HBsAg *hepatitis B surface antigen, *HBcAb* hepatitis B core antigen, *HCV* hepatitis C virus, *ALT* alanine transaminase, *GGT* Gamma-Glutamyltransferase, *AFP* α-fetoprotein, *AJCC* the American Joint Committee on Cancer, *TNM* tumor-node-metastasis

IER3 expression was assessed via immunohistochemistry (IHC). Briefly, paraffin-embedded tissue Sects. (5 μm thick) were deparaffinized, rehydrated, and treated for 15 min with 10 mM citric acid buffer at 100 °C to facilitate antigen retrieval. Sections were then probed overnight with primary anti-IER3 (Abcam, Cambridge, CA) at 4 °C, after which they were washed with PBS and probed for 30 min with an appropriate secondary antibody at 37 °C. After an additional PBS wash, sections were incubated for 2 min with DAB, followed by hematoxylin counterstaining. Slides were then evaluated by three pathologists blinded to patient clinical data. IER3 expression was evaluated by these pathologists based upon staining intensity (scored from 1–4) and on the percentage of IER3-positive cells. Scores ranged from 1 to 4 (1 for 0%, 2 for 1–10%, 3 for 11–50% and 4 for > 50%). The amount of these scores was employed to classify the specimens into two groups: 1–2 scores were considered “low” expression, whereas 3–4 scores were considered “high” expression.

### Statistical analysis

All data were analyzed using SPSS v.18.0, while GraphPad Prism 6.0 was employed for figure preparation. Chi-squared tests were used to assess relationships between patient clinicopathological findings and IER3 expression. Spearman’s correlation analysis was used to compare associations among analyzed variables. The Kaplan–Meier approach was used to prepare survival curves for the present study, with the log-rank test being employed to assess survival outcome significance. Data are means ± SD, and were compared via Student’s t-test or Tukey’s post hoc test where appropriate. P < 0.05 was the significance threshold.

## Results

### Assessment of HCC cell TRAIL sensitivity

**Fig. 1:**
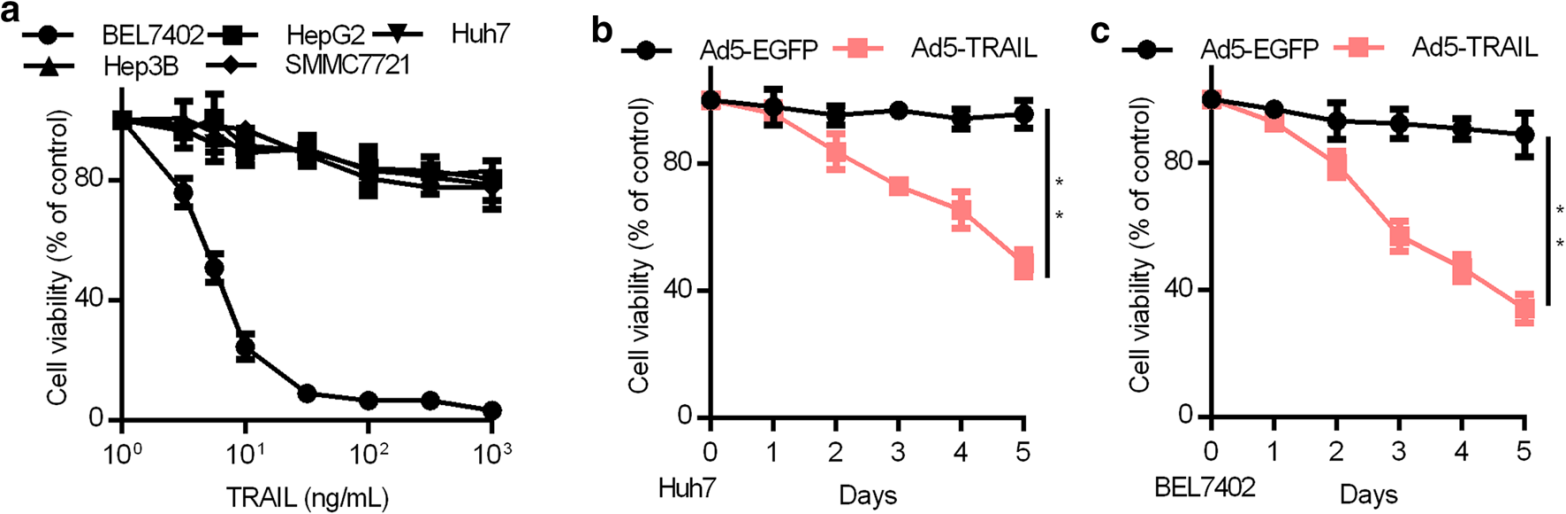
TRAIL inhibited cell growth in HCC cells. **a** BEL7402, HepG2, Huh7, Hep3B, and SMMC7721 cells were treated for 96 h using the indicated doses of recombinant human TRAIL, after which growth was assessed via MTT assay. Data are means ± SD of triplicate samples. Results are the average of three experiments. **P < 0.01, Dunnet’s post-hoc test. **b**, **c** BEL7402 and Huh7 cells were administered the indicated Ad5-TRAIL doses for the indicated time periods. **P < 0.01

To examine TRAIL-induced inhibition of HCC proliferation, we evaluated using an MTT assay. Briefly, we treated available HCC lines (BEL7402, HepG2, Huh7, Hep3B, and SMMC7721) with a range of doses of soluble TRAIL for a 96 h period revealing variable TRAIL sensitivity among these cell lines (Fig. 1a). Only the BEL7402 cells were found to be particularly sensitive to TRAIL administration in this context, with the other cell lines all exhibiting some degree of resistance. However, when cells were instead transduced with an adenovirus harboring TRAIL (Ad5-TRAIL), the Huh7, BEL7402, and SMMC7721 cell lines were all found to be sensitive to TRAIL-induced cell growth inhibition in a time-dependent fashion (P < 0.01) (Fig. 1a and Additonal file 1: Fig. S1a).

### TRAIL interacts with IER3

**Fig. 2:**
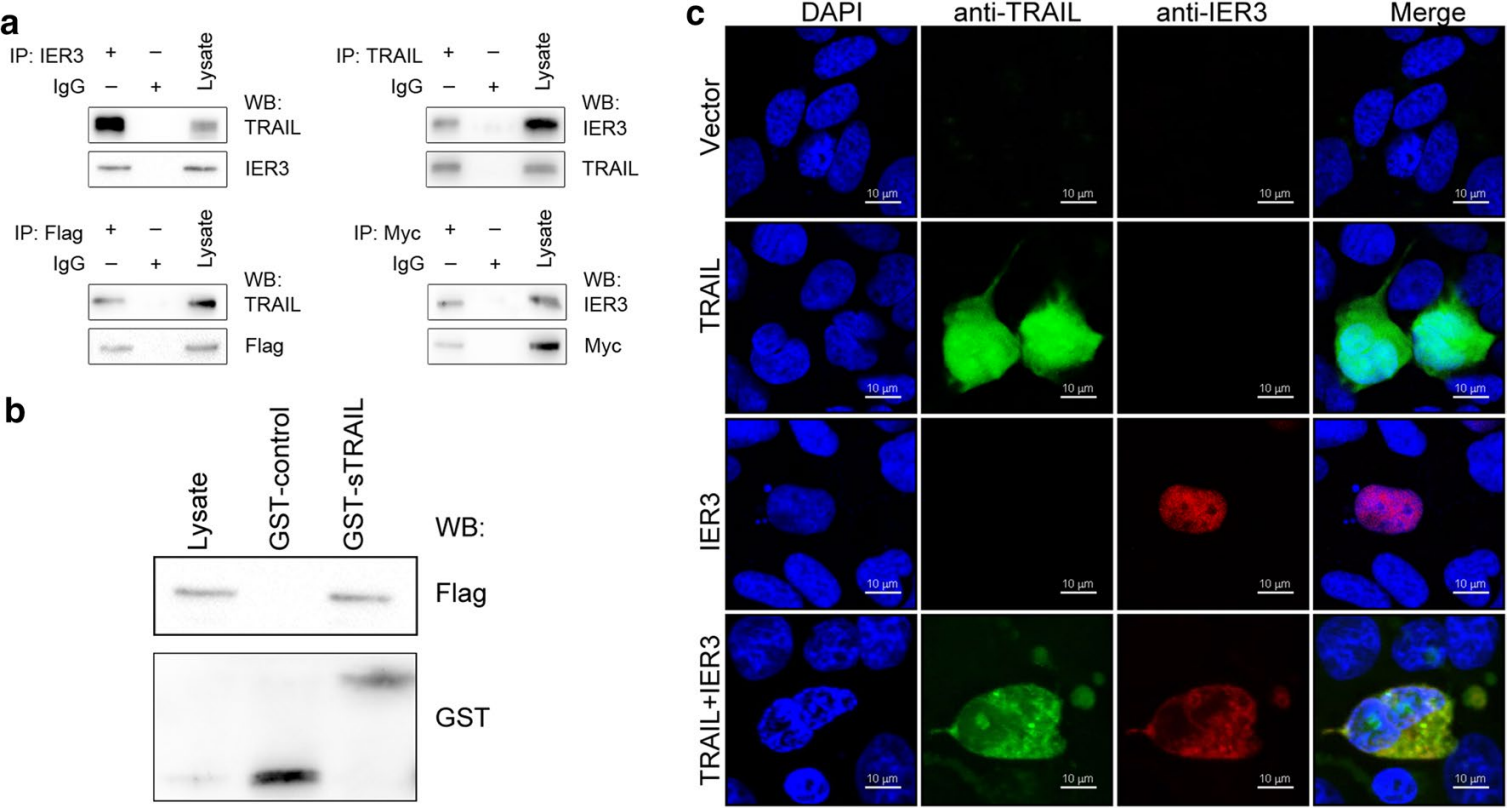
TRAIL interacts with IER3. **a** Huh7 cells were co-transfected plasmids encoding Myc-TRAIL and Flag-IER3, after which IER3- or Flag antibody-conjugated beads were used for co-immunoprecipitation (co-IP) analyses of lysates from these transfected cells. Western blotting was then used to detect TRAIL and IER3 in these samples using appropriate antibodies specific for TRAIL, IER3, Flag, or Myc. For reverse co-IP assays, cells were transfected as above, and then lysates were immunoprecipitated using anti-Myc or anti-TRAIL antibodies, after which TRAIL and IER3 levels in precipitates were measured as above. **b** Interactions between GST-TRAIL and IER3 were demonstrated in a GST pulldown assay. **c** TRAIL-Myc and IER3-Flag localization within Huh7 cells were assessed by fixing cells 24 h post-transfection, permeabilizing cells, and staining with antibodies specific for TRAIL (green) and IER3 (red), with DAPI (blue) being used to stain nuclei. Cells were then analyzed via confocal microscopy. Scale bars = 10 μm

This mode of delivery-dependent variability in TRAIL sensitivity suggests that the direct intracellular delivery of TRAIL via an adenoviral vector may bypass cell surface death receptors, enabling this protein to instead induce apoptosis via interacting with alternative protein targets. To identify potential intracellular TRAIL-interacting proteins, we utilized the NCBI database (https://www.ncbi.nlm.nih.gov/gene/8743) to identify proteins that can interact with TRAIL. This analysis identified 37 putative interaction targets, including HDAC1, SP1, and RIPK1. To further assess the existence of potential functional associations among these proteins, we queried the STRING (http://string-db.org/) database of known and predicted protein–protein interactions. This analysis revealed that TRAIL can interact with IER3 (Additional file 1: Fig. S1b), indicating that this interaction may be important as a regulator of the survival and proliferative activity of HCC cells. IER3 can regulate a range of processes such as DNA repair, inflammation, proliferation, and apoptosis [21, 26, 27]. In order to confirm that TRAIL can interact with IER3, we next conducted a co-IP assay. Briefly, we first generated IER3-Flag and TRAIL-Myc vectors (Additional file 2: Fig. S2a), after which we directly assessed and confirmed the existence of TRAIL/IER3 interactions within HCC cells (Fig. 2a). We also employed a glutathione S-transferase (GST) pull-down assay approach (Additional file 2: Fig. S2b, c), which further confirmed that GST-TRAIL was capable of interacting with IER3 (Fig. 2b). Immunofluorescent staining of Huh7 and SMMC7721 cells also demonstrated the ability of TRAIL (green) and IER3 (red) to colocalize with one another (Fig. 2c and Additional file 2: Fig. S2d). Together, these findings confirmed that TRAIL can interact with IER3 within HCC cells.

### TRAIL and IER3 suppress HCC cell migration and induce apoptotic cell death

**Fig. 3:**
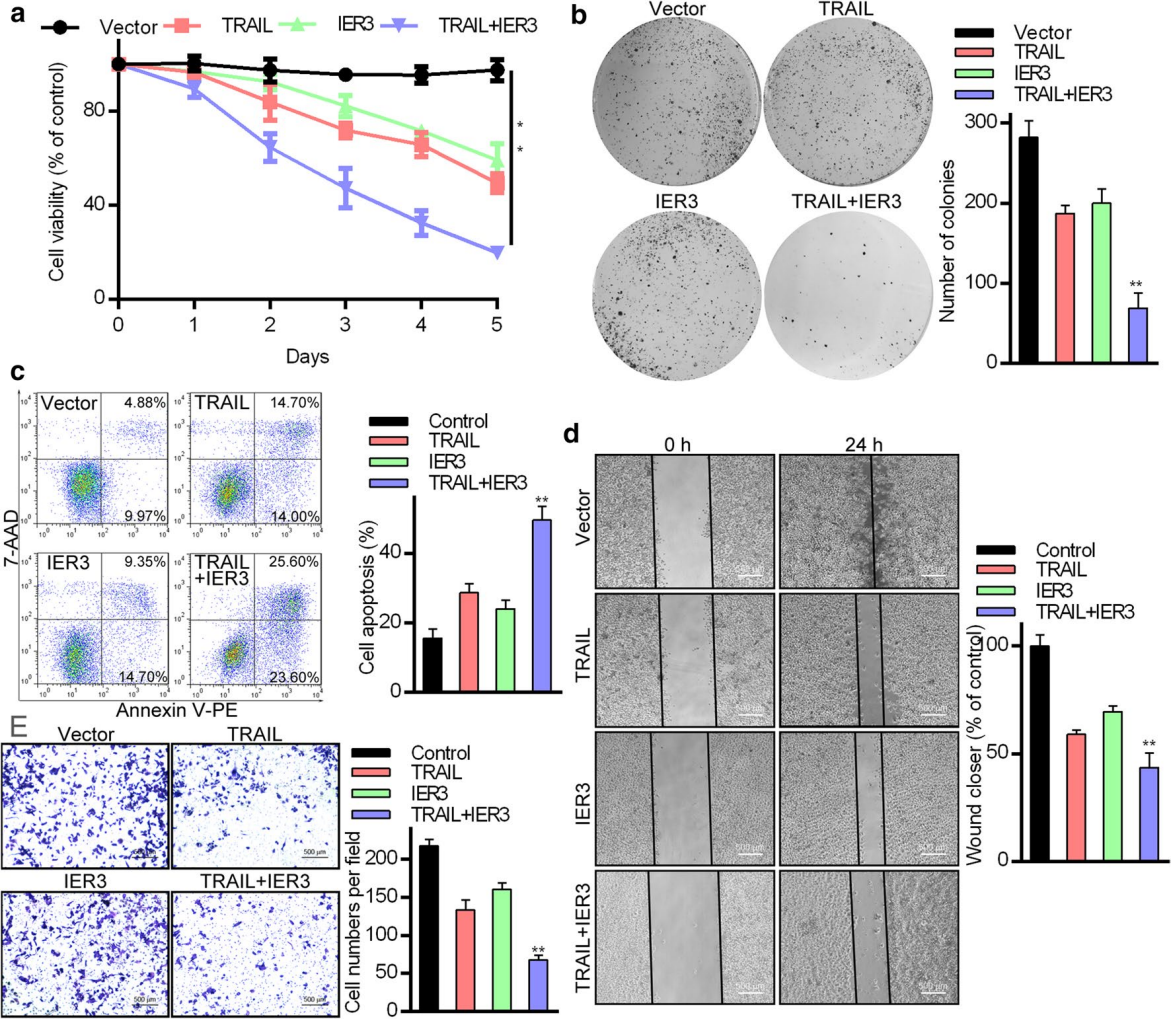
TRAIL and IER3 suppress HCC cell survival, migration, and colony formation. **a** Huh7 cells were co-infected with Ad5-TRAIL and Ad5-IER3, after which viability was assessed via MTT assay. **b** A colony formation assay was employed to assess the viability of cells prepared as in **a**. **c** The impact of TRAIL and IER3 synergistic effect on apoptotic cell death in Huh7 cells was evaluated via flow cytometry. **d** In a wound healing assay, adenoviral of TRAIL and IER3 synergistic effect was found to suppress Huh7 cell migration. **e** A Transwell assay was used to assess the migration of cells that had been infected with Ad5-TRAIL or/and Ad5-IER3. Data are means ± s.d. (*P < 0.05, **P < 0.01)

To evaluate the functional impact of TRAIL/IER3 interactions within HCC cells, we next infected Huh7 and SMMC7721 cell lines with these adenoviral vectors as above and confirmed the efficiency of this infection approach (Additional file 2: Fig. S2e). MTT and colony formation assays were then used to evaluate these cells, revealing that TRAIL and IER3 synergistic effect was associated with reduced viability for both of these cell lines (Fig. 3a, b and Additional file 3: Fig. S3a). An Annexin V-PE/7-AAD dual-staining approach additionally revealed that TRAIL/IER3 synergistic effect induced apoptotic Huh7 and SMMC7721 cell death more readily than did control adenoviral infection (Fig. 3c and Additional file 3: Fig. S3b). In line with these results, we also found TRAIL/IER3 synergistic effect to suppress HCC cell migratory activity in Transwell and wound healing assays (Fig. 3d, e). These findings suggest that TRAIL and IER3 can promote the apoptotic death of HCC cells and can additionally impair the migratory activity of these cells.

### TRAIL and IER3 inhibit in vivo HCC cell growth

**Fig. 4:**
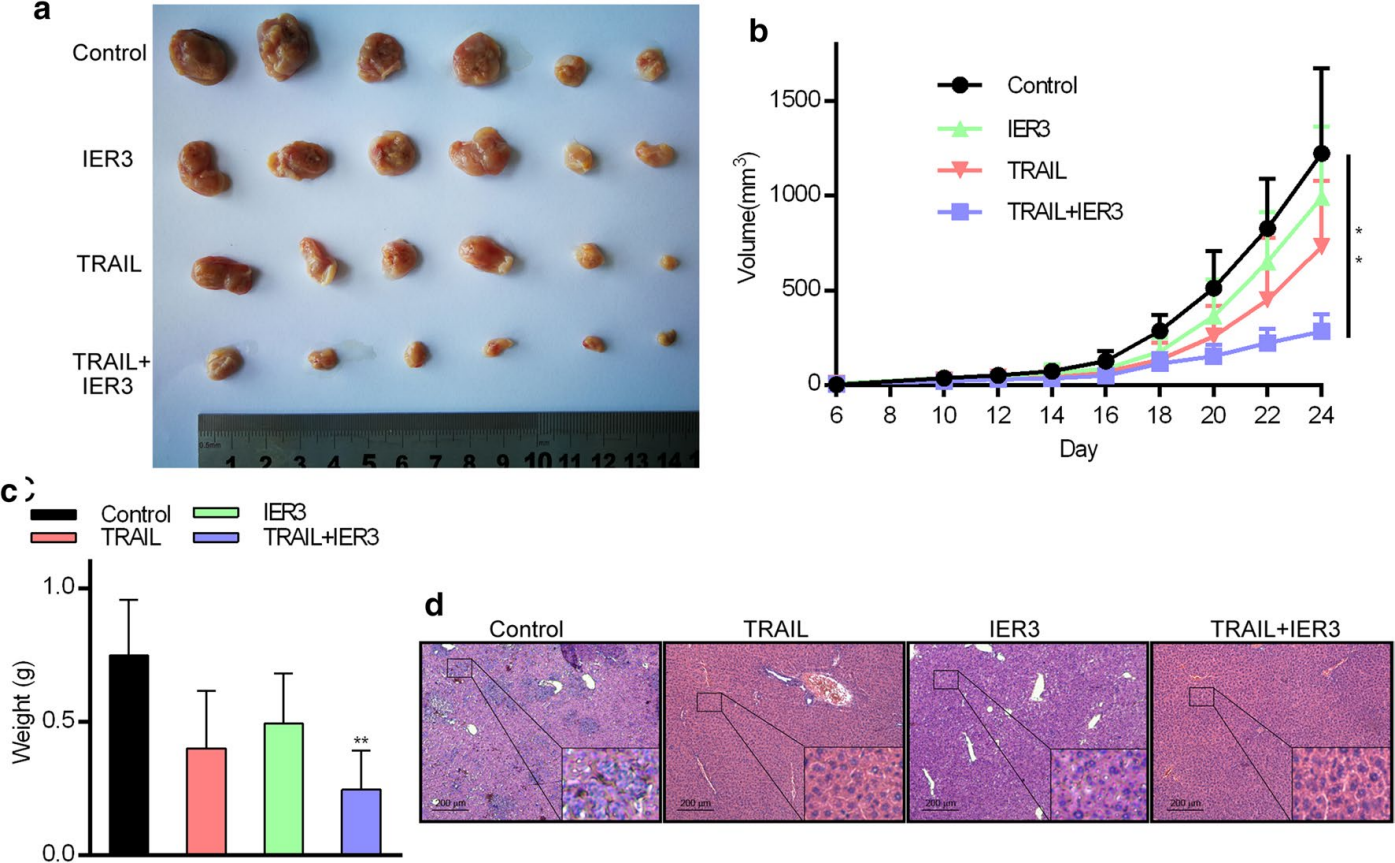
TRAIL/IER3 synergistic effect inhibits in vivo xenograft Huh7 tumor growth. **a** On day 24, animals were euthanized and tumors were imaged. **b** Tumor growth over the 24-day study period. **c** Tumor weights. **d** H&E staining was used to evaluate tumor histology. Scale bar = 200 mm. **P < 0.01

We next assessed the ability of TRAIL/IER3 synergistic effect in HCC cells to impact in vivo tumor growth. Briefly, we established an HCC xenograft model by subcutaneously implanting Huh7 cells into athymic nude mice. After 24 days, we found that tumors in which TRAIL and IER3 had been overexpressed were significantly smaller than control tumors in these animals (Fig. 4a, c), consistent with observed differences in tumor growth curves between groups (Fig. 4b). At this day 24 time point, we additionally evaluated histopathological findings in these mice and found that TRAIL/IER3 synergistic effect in these tumors was associated with significant reductions in liver metastasis relative to control tumors (Fig. 4d). Comparable findings were also made when mice were instead subcutaneously implanted with SMMC7721 cells (Additional file 4: Fig. S4). These results thus suggest that TRAIL and IER3 upregulation in HCC cells can impair tumor growth and metastasis in vivo*.*

### TRAIL and IER3 inhibit Wnt target gene expression

**Fig. 5:**
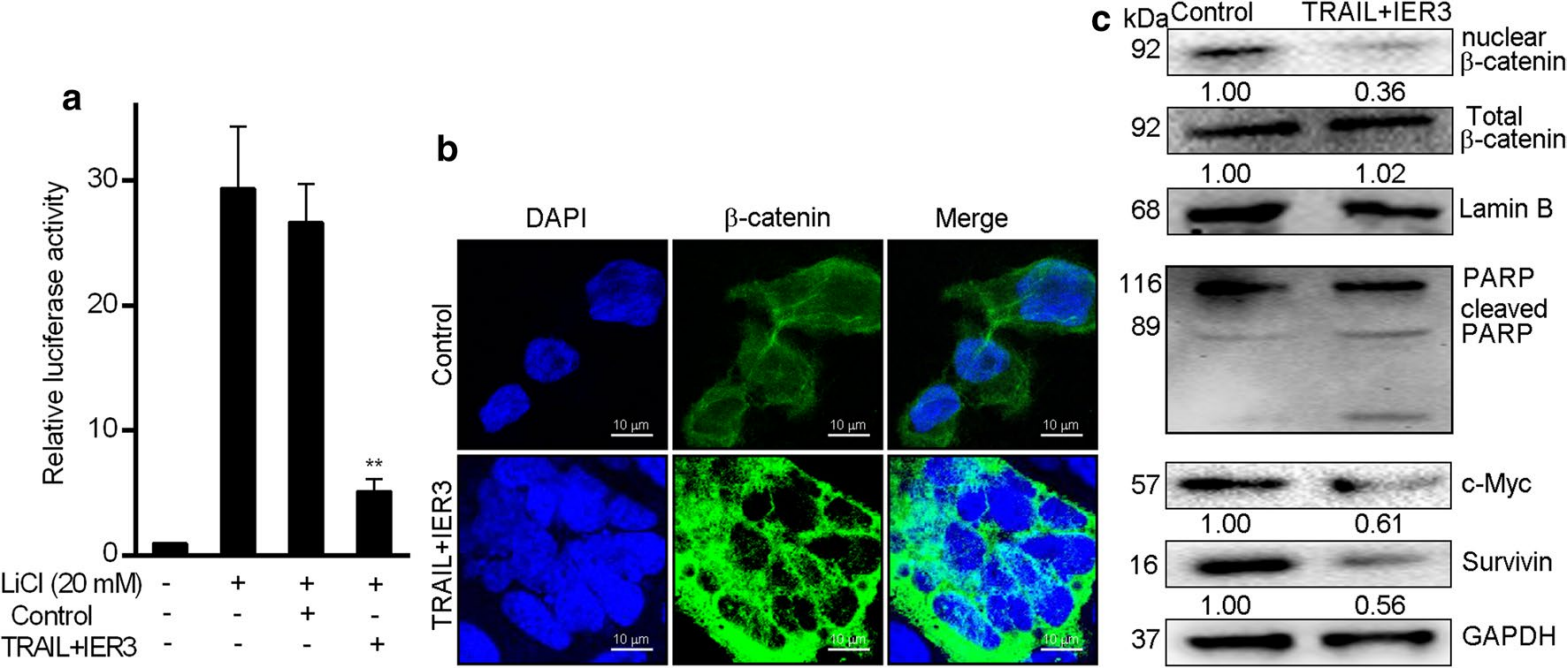
TRAIL/IER3 inhibit Wnt/β-catenin signaling. **a** After stimulation with LiCl (20 mM), TOP/FOP ratio in a luciferase reporter assay revealed that TRAIL/IER3 synergistic effect impacted Wnt signaling in Huh7 cells. Renilla luciferase activity was used to normalize firefly luciferase activity for all samples, with experiments being repeated three times. **P < 0.01. **b**, **c** Immunofluorescence and Western blotting analysis were used to assess nuclear β-catenin, PARP, c-Myc, and survivin levels in Huh7 cells following TRAIL/IER3 synergistic effect. Scale bar = 10 µm

Previously, we have discovered that Wnt/β-catenin signaling is activated to a greater extent in HCC cells than in normal liver cells [21]. Therefore, we hypothesized that TRAIL/IER3 might target HCC by inhibiting Wnt/β-catenin signaling. To assess the impact of TRAIL/IER3 to influence Wnt/β-catenin pathway activation, we examined the impact of these proteins in cells that had been stimulated with lithium chloride (LiCl), which is known to activate Wnt/β-catenin signaling [28]. To measure Wnt/β-catenin signaling activity in this assay context, we assessed the relative luciferase activity of TOPflash and FOPflash reporters (the TOP/FOP ratio) [29]. Briefly, we transfected Huh7 cells with the appropriate reporters, and then treated them for 24 h with LiCl (20 mM), after which cells were infected with Ad5-TRAIL and Ad5-IER3 or Ad5-EGFP. LiCl treatment was confirmed to increase the TOP/FOP ratio of these HCC cells (P < 0.01), consistent with Wnt/β-catenin signaling pathway activation. Relative to the LiCl-treated control cells, those cells that had been infected with Ad5-TRAIL and Ad5-IER3 exhibited a significant decrease in TOP/FOP ratio values consistent with the suppression of Wnt/β-catenin signaling (P < 0.05) (Fig. 5a). To further assess the ability of TRAIL/IER3 to suppress Wnt signaling, we additionally evaluated β-catenin localization within cells via immunofluorescence. This approach indicated that TRAIL/IER3 synergistic effect was associated with reduced nuclear β-catenin expression (Fig. 5b). Consistent with these findings, when we employed a Western blotting approach to analyze these cells, we found that TRAIL/IER3 synergistic effect were associated with decreases in nuclear β-catenin, survivin, and c-Myc protein levels, and with increased PARP expression (Fig. 5c).

### IER3 downregulation correlates with HCC prognosis

**Fig. 6:**
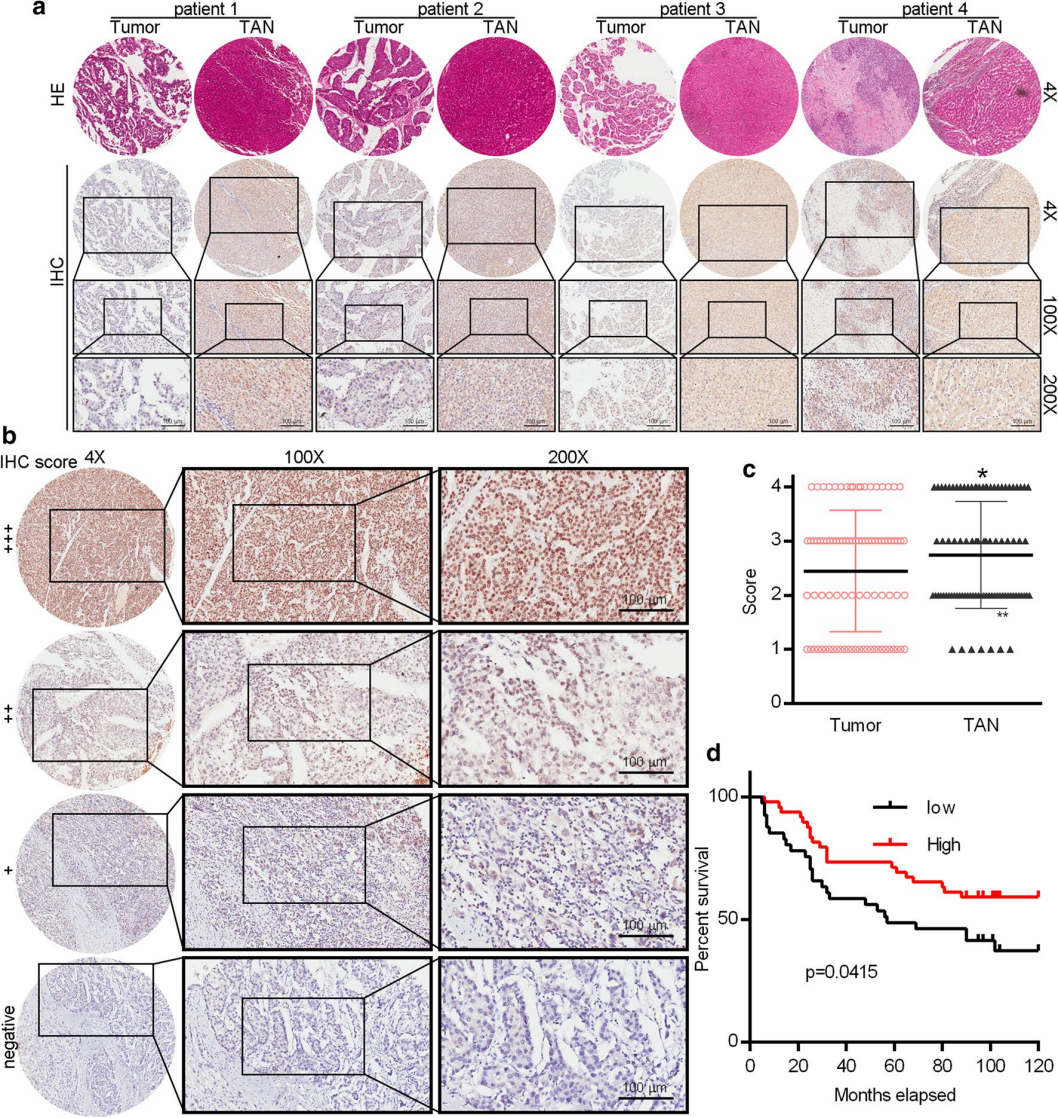
IER3 expression is related to HCC prognosis. **a** IER3 expression in primary HCC tissue arrays was assessed via IHC, with representative images of tumors in different staining categories and adjacent normal tissue samples being shown. H&E and IHC staining were conducted by Shanghai Outdo Biotech Co., Ltd. (Shanghai, China), and were used to evaluate tumor area. **b** IHC scores were determined based upon the percentage of IER3-positive cells as a fraction of total tumor cells. Staining intensity was scored from 1–4, with scores of 1–2 indicating low IER3 expression and scores of 3–4 indicating high expression. Scale bars = 100 μm. **c** IER3 expression in matched primary tumor and tumor adjacent normal (TAN) tissue samples from HCC patients was evaluated via IHC. **d** The overall survival (OS) of 90 HCC patients with high (score: 3–4) and low (score: 1–2) IER3 IHC staining scores was assessed via the Kaplan–Meier method. *P < 0.05

IER3 has been shown to control apoptotic cell death in many different physiological contexts [26, 30–32]. In order to assess how IER3 expression is associated with HCC patient survival, we examined the expression of this protein via IHC in HCC patient tumor microarrays (TMAs). In total, we analyzed 90 HCC patient tumor and tumor adjacent normal (TAN) tissue samples (Fig. 6a). IER3 staining intensity for these samples was scored from 1–4: 1 (negative), 0% positive; 2 ( +), 1–10% positive; 3 (+ +), 11–50% positive; 4 (+ + +), > 50% positive (Fig. 6b). In addition, clinicopathological data pertaining to the patients included in this TMA were compiled, including age, gender, HBsAg, HBcAb, HCV, liver cirrhosis, total bilirubin level, ALT level, GGT level, cirrhotic nodules, vascular invasion, tumor number, tumor size, recrudescence, and AJCC TNM stage (Table 1). Patients included in this analysis were between the ages of 31 and 78 (mean: 52.37 ± 10.76 years). We analyzed IER3 expression levels in HCC tissues and tumor adjacent normal (TAN) tissues, and IHC analysis revealed that IER3 expression was undetectable or found to be only expressed at low levels in HCC cases compared with TAN (Fig. 6c). When patients were stratified into IER3-high or -low groups based upon IHC staining scores, a Kaplan–Meier survival analysis indicated that IER3-high patients had a significantly longer overall survival (OS) relative to IER3-low patients (71.43% vs. 38.09%) (Fig. 6d). This suggests that IER3 upregulation may be associated with a better prognosis in HCC patients.

The relationship between IER3 expression and HCC patient clinicopathological features was additionally assessed. We observed a strong relationship between IER3 expression and GGT level (P = 0.030), tumor size (P < 0.001), and recrudescence (P = 0.047). In contrast, no relationship between IER3 expression and age (P = 0.219), gender (P = 0.708), or other clinicopathological features was evaluated (Table 2). We additionally evaluated the relationship between IER3 expression and HCC prognosis via a Cox regression analysis approach, which revealed that IER3 (P = 0.041), GGT level (P = 0.034), and AJCC TNM stage (P = 0.021) were all significantly associated with HCC patient survival in univariate analyses (Table 3). Subsequent multivariate Cox regression analysis revealed that gender (P = 0.048), HBsAg (P = 0.037), GGT (P = 0.017), and cirrhotic nodules (P = 0.033) were all predictors of HCC patient survival (Table 3). Together, these results suggest that IER3 expression and other clinicopathological features are associated with HCC patient prognosis.

**Table 2:** Spearman analysis of correlation between TUG1 and clinicopathological

Variables	IER3 expression level	
	Spearman correlation	p value
Age (years, < 50 vs. ≥ 50)	− 0.129	0.224
Gender (male/female)	0.039	0.712
HBsAg (positive/negative)	0.179	0.094
HBcAb (positive/negative)	0.151	0.163
HCV (positive/negative)	0.099	0.359
Liver cirrhosis (yes/no)	− 0.086	0.425
Total bilirubin (mmol/L, < 21 vs. ≥ 21)	0.09	0.403
ALT (U/L, < 60 vs. ≥ 60)	− 0.179	0.094
GGT (U/L, < 40 vs. ≥ 40)	− 0.23	*0.03*
Cirrhotic nodules (single/multiple)	0.032	0.768
Vascular invasion (yes/no)	− 0.14	0.219
Tumor number (single/multiple)	0.001	0.994
Tumor size (cm, < 5 vs. ≥ 5)	− 0.414	* < 0.001*
Recrudescence (yes/no)	− 0.21	*0.047*
AJCC TNM stage (I + II vs III + IV)	− 0.079	0.461

Italics numbers indicate significant differences (P < 0.05)

*HCC* hepatocellular carcinoma, *HBsAg* hepatitis B surface antigen, *HBcAb* hepatitis B core antigen, *HCV *hepatitis C virus, *ALT* alanine transaminase, *GGT* Gamma-Glutamyltransferase, *AFP* α-fetoprotein, *AJCC* the American Joint Committee on Cancer, *TNM* tumor-node-metastasis

**Table 3:** Univariate and multivariate analyses of various prognostic parameters in patients with HCC Cox-regression analysis

	Univariate analysis			Multivariate analysis		
	p value	Hazard Ratio	95% confidence interval	p value	Hazard Ratio	95% confidence interval
IER3	*0.041*	0.549	0.305–0.989	0.046252	0.596	0.246–1.444
Gender (male/female)	0.241	0.496	0.153–1.601	*0.048*	0.168	0.029–0.983
HBsAg (positive/negative)	0.906	1.045	0.502–2.175	*0.037*	0.276	0.082–0.924
GGT (U/L, < 40 vs. ≥ 40)	*0.034*	2.148	1.059–4.356	*0.017*	4.274	1.300–14.054
Cirrhotic nodules (single/multiple)	0.121	3.077	0.745–12.713	*0.033*	12.069	1.216–119.809
AJCC TNM stage (I + II vs III + IV)	*0.021*	4.115	1.237–13.688	0.857	0.792	0.062–10.152

Italics numbers indicate significant differences (P < 0.05)

*HCC* hepatocellular carcinoma, *HBsAg* hepatitis B surface antigen, *GGT* Gamma-Glutamyltransferase, *AJCC* the American Joint Committee on Cancer, *TNM* tumor-node-metastasis

## Discussion

HCC accounts for over 90% of liver cancer cases globally, making it one of the most prevalent forms of malignancy in humans [33], with roughly 841,080 new cases and 781,631 deaths worldwide each year [3]. Despite therapeutic advances, HCC patients still have a poor 5-year survival rate owing to high rates of tumor metastasis and recurrence [34]. It is thus vital that the molecular mechanisms governing human HCC be better understood such that it can be more effectively treated.

TRAIL is a type 2 transmembrane protein [35] that can trigger apoptotic cell death [36–38]. TRAIL-induced apoptosis occurs following binding to the DR4 and DR5 receptors, with DR4 binding being thought to be the primary driver of apoptosis [39]. TRAIL binding to these death receptors results in FADD recruitment, which in turn triggers caspase-8 activation and downstream apoptotic signaling [40–42], although the efficacy of such signaling is dependent upon the glycosylation status of DR4 [43, 44] and DR5 [45, 46], and on TRAIL multimerization [47–49]. Recent reviews have assessed the potential utility of TRAIL as an anti-tumor agent [35], and the delivery of TRAIL to tumors using alginate-based nanocomposites [50], carbon nanotubes [51], and other inorganic and organic nanoparticles has also been explored [52, 53]. Adenoviral particles harboring TRAIL have also been shown to be capable of triggering tumor cell apoptosis. However, these prior studies have only evaluated the impact of TRAIL binding to cell surface receptors on apoptotic signaling, and have not explored intracellular mechanisms whereby TRAIL can induce apoptotic cell death.

IER3 is an early response gene family member that is important as a regulator of cellular proliferation and differentiation [54]. It has also been shown to regulate apoptosis in a cell- and stimulus-dependent fashion such that it can either be pro- or anti-apoptotic in specific contexts [23]. Elevated IER3 expression is commonly detected in a range of tumors with a poor prognosis including bladder, breast, pancreatic, and melanoma tumors [54, 55]. However, there is also evidence that IER3 can promote the apoptotic death of HeLa and 293 tumor cells under serum starvation conditions [26, 56]. The factors that determine whether IER3 functions in a pro- or anti-apoptotic manner in a given context remain to be fully elucidated [26].

To evaluate the mechanisms whereby TRAIL impacts HCC malignancy, we herein evaluated the interactions between TRAIL and other proteins in tumor cells. Through co-IP and immunofluorescence assays, we demonstrated that TRAIL can interact with IER3 in the cytoplasm and nucleus of HCC cells. We further found that TRAIL and IER3 can induce apoptotic cell death and can suppress the migration and proliferation of these HCC cells. Indeed, in MTT assays we found that TRAIL/IER3 synergistic effect was associated with reduced HCC cell viability relative to the overexpression of TRAIL or IER3 alone. This was consistent with Annexin V/PI staining results and migration assays, which revealed that TRAIL/IER3 synergistic effect was associated with significantly enhanced apoptotic death and impaired migration as compared to the overexpression of TRAIL or IER3 alone. We additionally observed differences in Wnt pathway activation and target gene expression in HCC cells following TRAIL/IER3 synergistic effect, suggesting that these proteins can inhibit Wnt pathway signaling. Together, these findings suggest that TRAIL/IER3 serves as a critical protein–protein signaling complex in HCC cells.

## Conclusions

The results of the present study demonstrate that TRAIL is able to directly interact with IER3, and that both of these proteins are important inhibitors of HCC progression. TRAIL and IER3 synergistic effect can inhibit the proliferation and migratory activity of HCC cells, while also inducing their apoptotic death. This TRAIL/IER3 axis may therefore be a viable therapeutic target for the treatment of HCC, although further research will be necessary to validate this hypothesis and to evaluate the molecular mechanisms whereby these proteins govern HCC onset and progression.

## Supplementary Information

**Additional file 1: Fig. S1.** The impact of TRAIL-expression on HCC cells.** a** SMMC7721 cells were infected with Ad5-TRAIL at the indicated doses for a range of time periods. **P < 0.01.** b** Functional associations among TRAIL target genes. All 37 of these putative interacting genes were uploaded into the STRING database (http://string-db.org/), and TRAIL-related protein interaction networks were then evaluated.

**Additional file 2: Fig. S2.** TRAIL interacts with IER3.** a** IER3-Flag and TRAIL-Myc vector maps.** b** IER3-Flag and GST-TRAIL vector maps.** c** The GST-TRAIL fusion protein was purified and precipitated using glutathione-agarose via SDS-PAGE with gel staining.** d** TRAIL (green) and IER3 (red) colocalization in SMMC7721 cells was assessed via immunofluorescent staining. Scale bar = 10 μm.** e** Ad5-TRAIL and Ad5-IER3 infection of Huh7 and SMMC7721 cells. Scale bar = 100 μm.

**Additional file 3: Fig. S3.** TRAIL and IER3 suppress HCC cell survival, migration, and colony formation.** a** SMMC7721 cells were co-infected with Ad5-TRAIL and Ad5-IER3, after which a colony formation assay was employed to assess the viability of these cells.** b** The impact of TRAIL and IER3 synergistic effect on apoptotic cell death in SMMC7721 cells was evaluated via flow cytometry.** c** In a wound healing assay, adenoviral overexpression of TRAIL and IER3 was found to suppress SMMC7721 cell migration.** d** A Transwell assay was used to assess the migration of cells that had been infected with Ad5-TRAIL or/and Ad5-IER3. Data are means ± sd. (*P < 0.05, **P < 0.01).

**Additional file 4: Fig. S4.** TRAIL and IER3 suppress HCC cell survival, migration, and colony formation.** a** SMMC7721 cells were co-infected with Ad5-TRAIL and Ad5-IER3, after which a colony formation assay was employed to assess the viability of these cells.** b** The impact of TRAIL and IER3 synergistic effect on apoptotic cell death in SMMC7721 cells was evaluated via flow cytometry.** c** In a wound healing assay, adenoviral overexpression of TRAIL and IER3 was found to suppress SMMC7721 cell migration.** d** A Transwell assay was used to assess the migration of cells that had been infected with Ad5-TRAIL or/and Ad5-IER3. Data are means ± sd. (*P < 0.05, **P < 0.01).

## Data Availability

The datasets generated and/or analyzed during the current study are available from the corresponding author on reasonable request.
