# Supplementary figures and images for: TRAIL promotes hepatocellular carcinoma apoptosis and inhibits proliferation and migration via interacting with IER3
Source: Cancer Cell Int. 2021 Jan 20;21:63. doi: 10.1186/s12935-020-01724-8 (PMC7816514; doi:10.1186/s12935-020-01724-8)

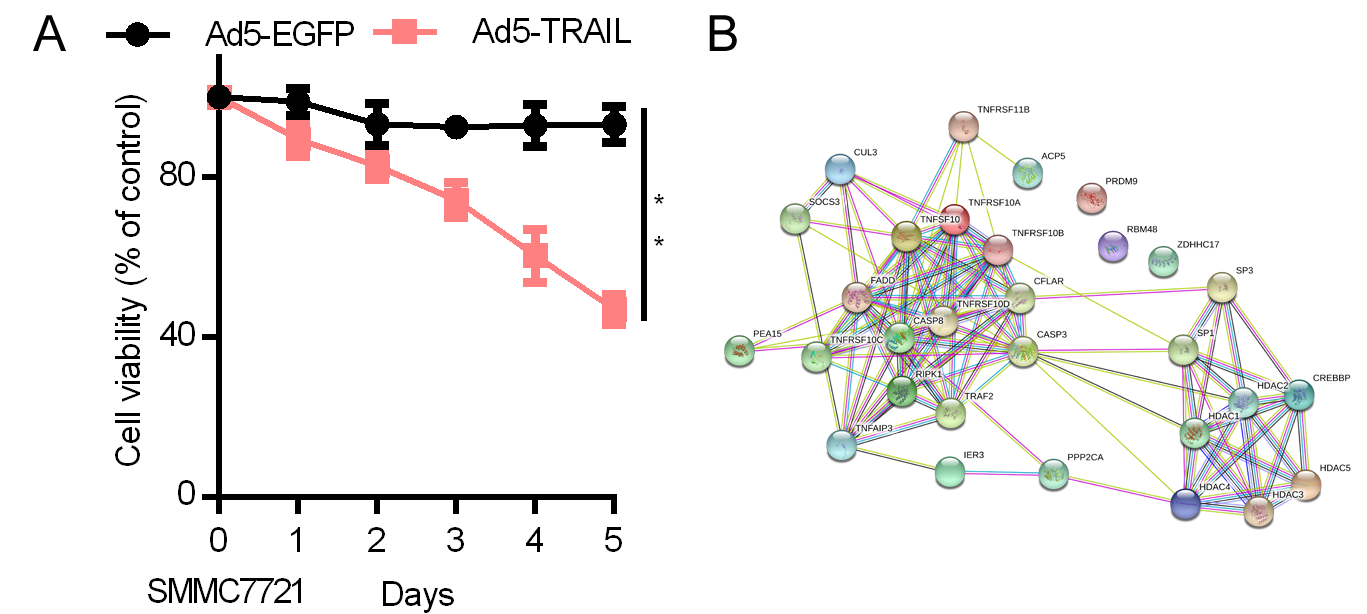

Supplement: Supplementary file 1 — Additional file 1: Fig. S1. The impact of TRAIL-expression on HCC cells. a SMMC7721 cells were infected with Ad5-TRAIL at the indicated doses for a range of time periods. **P < 0.01. b Functional associations among TRAIL target genes. All 37 of these putative interacting genes were uploaded into the STRING database (http://string-db.org/), and TRAIL-related protein interaction networks were then evaluated. [file 12935_2020_1724_MOESM1_ESM.tif]

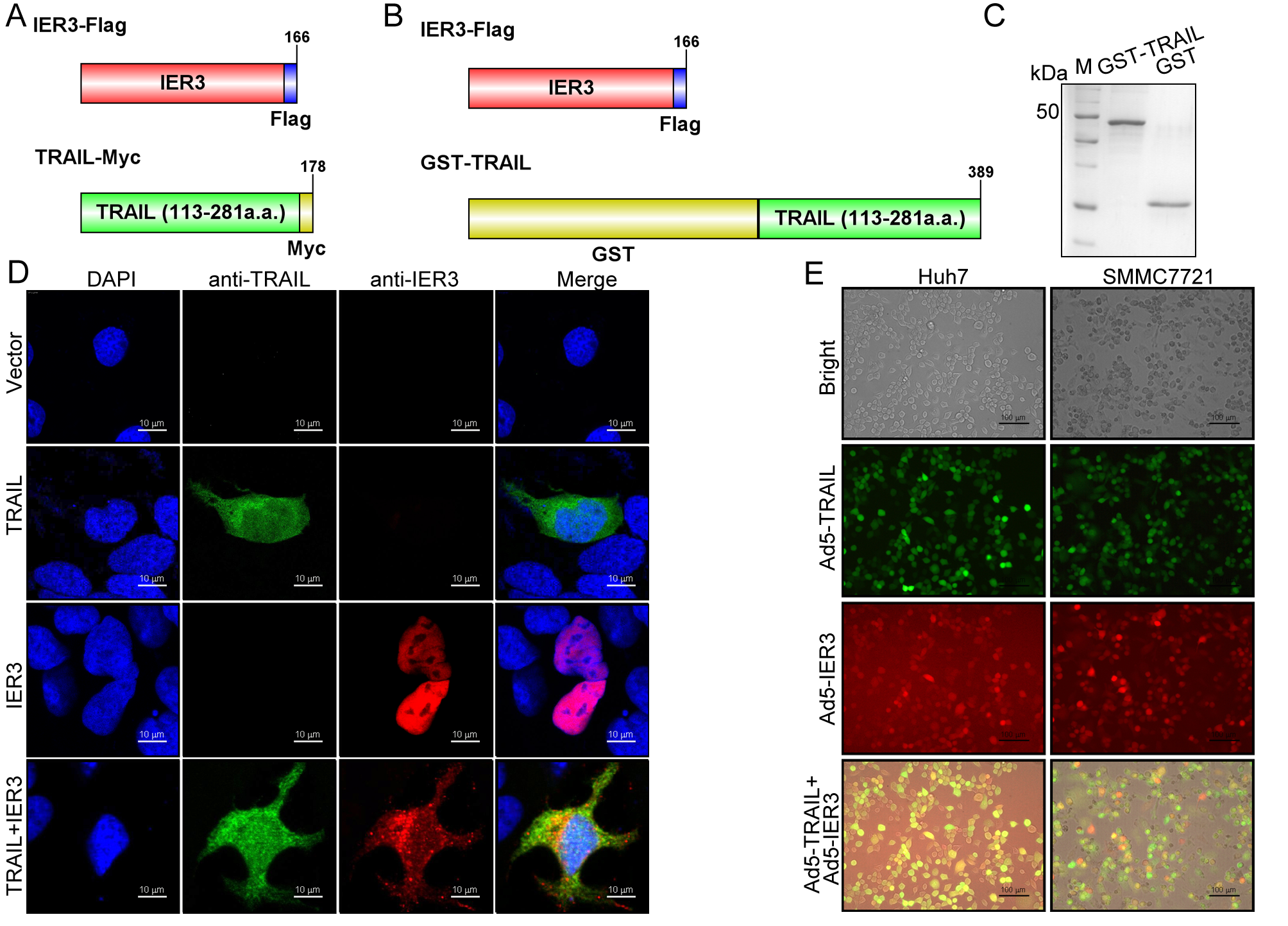

Supplement: Supplementary file 2 — Additional file 2: Fig. S2. TRAIL interacts with IER3. a IER3-Flag and TRAIL-Myc vector maps. b IER3-Flag and GST-TRAIL vector maps. c The GST-TRAIL fusion protein was purified and precipitated using glutathione-agarose via SDS-PAGE with gel staining. d TRAIL (green) and IER3 (red) colocalization in SMMC7721 cells was assessed via immunofluorescent staining. Scale bar = 10 μm. e Ad5-TRAIL and Ad5-IER3 infection of Huh7 and SMMC7721 cells. Scale bar = 100 μm. [file 12935_2020_1724_MOESM2_ESM.tif]

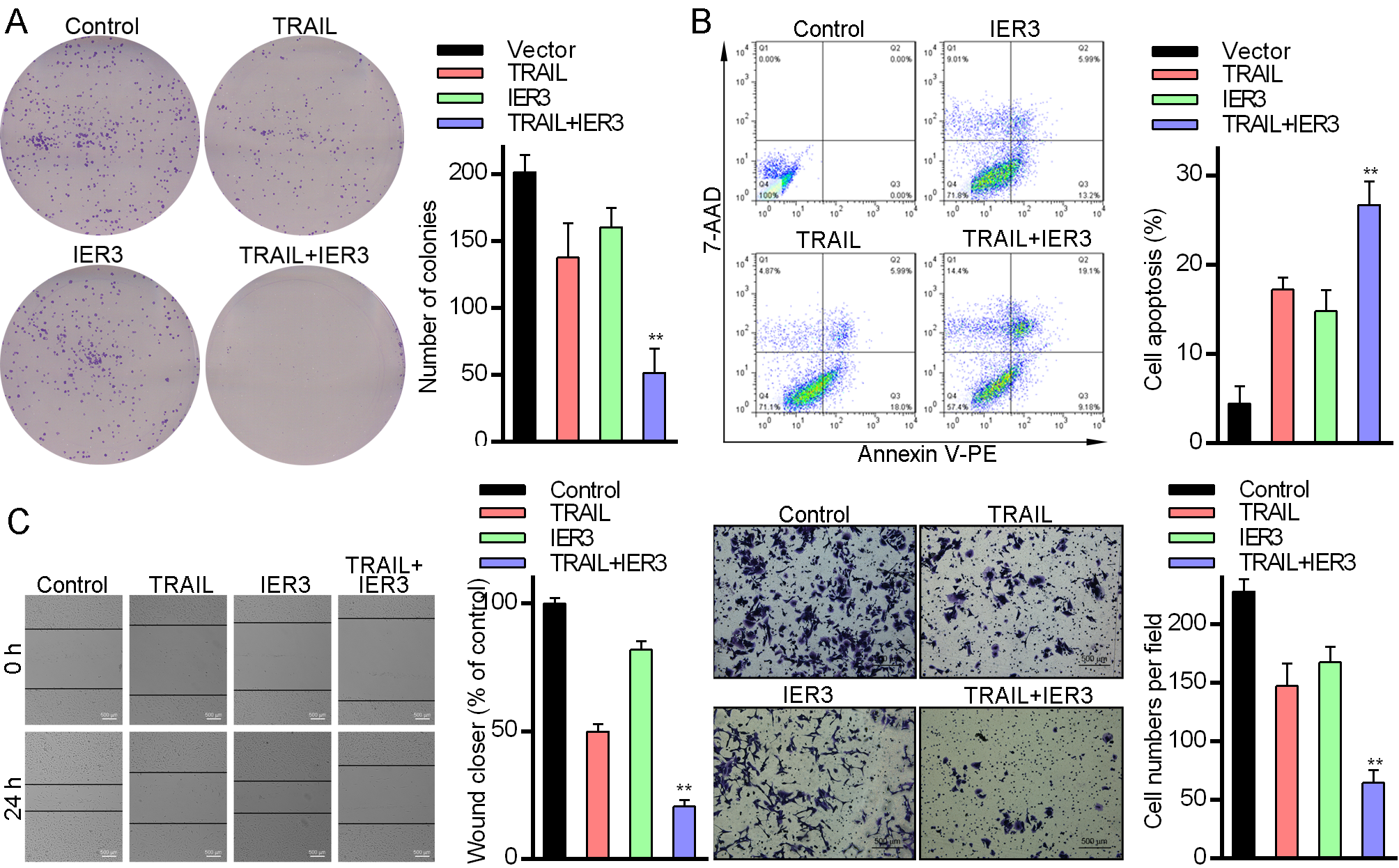

Supplement: Supplementary file 3 — Additional file 3: Fig. S3. TRAIL and IER3 suppress HCC cell survival, migration, and colony formation. a SMMC7721 cells were co-infected with Ad5-TRAIL and Ad5-IER3, after which a colony formation assay was employed to assess the viability of these cells. b The impact of TRAIL and IER3 synergistic effect on apoptotic cell death in SMMC7721 cells was evaluated via flow cytometry. c In a wound healing assay, adenoviral overexpression of TRAIL and IER3 was found to suppress SMMC7721 cell migration. d A Transwell assay was used to assess the migration of cells that had been infected with Ad5-TRAIL or/and Ad5-IER3. Data are means ± sd. (*P < 0.05, **P < 0.01). [file 12935_2020_1724_MOESM3_ESM.tif]

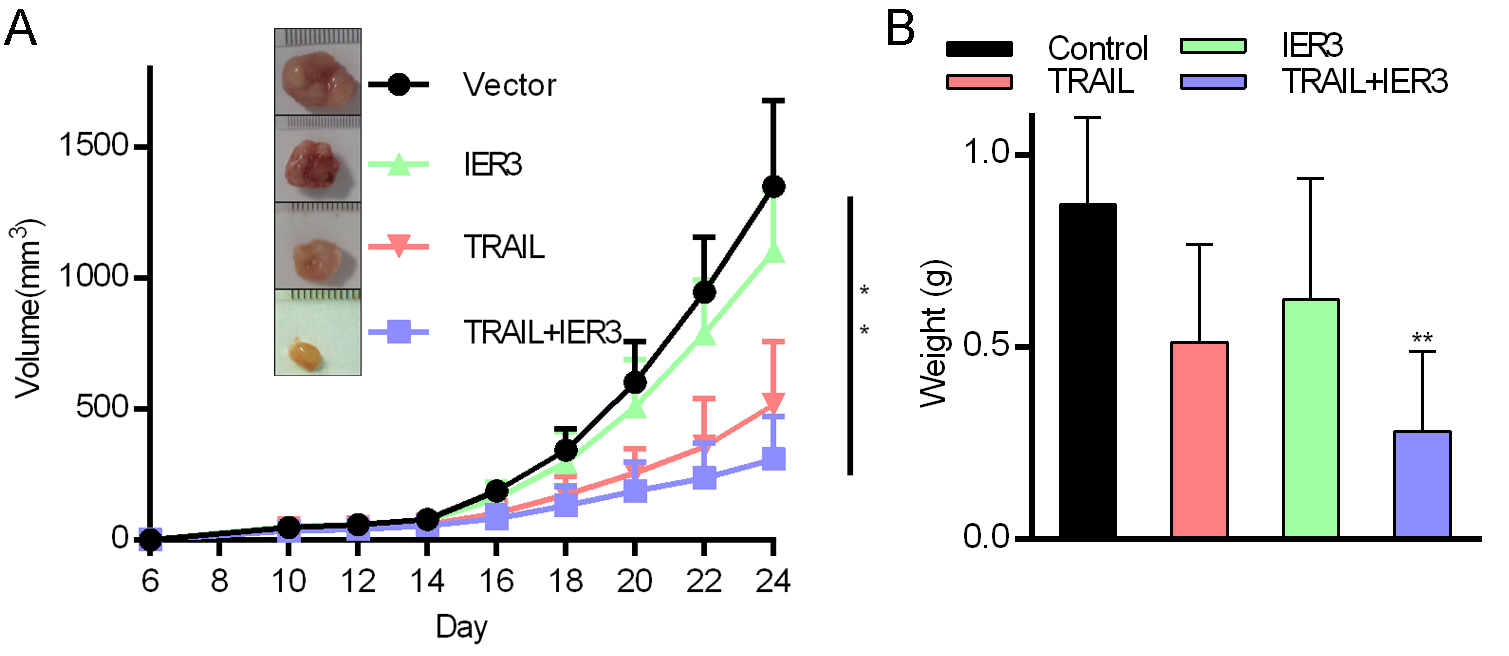

Supplement: Supplementary file 4 — Additional file 4: Fig. S4. TRAIL and IER3 suppress HCC cell survival, migration, and colony formation. a SMMC7721 cells were co-infected with Ad5-TRAIL and Ad5-IER3, after which a colony formation assay was employed to assess the viability of these cells. b The impact of TRAIL and IER3 synergistic effect on apoptotic cell death in SMMC7721 cells was evaluated via flow cytometry. c In a wound healing assay, adenoviral overexpression of TRAIL and IER3 was found to suppress SMMC7721 cell migration. d A Transwell assay was used to assess the migration of cells that had been infected with Ad5-TRAIL or/and Ad5-IER3. Data are means ± sd. (*P < 0.05, **P < 0.01). [file 12935_2020_1724_MOESM4_ESM.tif]
